# Endometriosis in Transgender Men: Bridging Gaps in Research and Care—A Narrative Review

**DOI:** 10.3390/biomedicines12071481

**Published:** 2024-07-04

**Authors:** Aris Kaltsas, Marios Stavropoulos, Evangelos N. Symeonidis, Zisis Kratiras, Athanasios Zachariou, Athanasios Zikopoulos, Efthalia Emmanouela Chrisofos, Fotios Dimitriadis, Nikolaos Sofikitis, Michael Chrisofos

**Affiliations:** 1 Third Department of Urology, Attikon University Hospital, School of Medicine, National and Kapodistrian University of Athens, 12462 Athens, Greece; ares-kaltsas@hotmail.com (A.K.); stamarios@yahoo.gr (M.S.); zkratiras@gmail.com (Z.K.); 2 Department of Urology II, European Interbalkan Medical Center, 55535 Thessaloniki, Greece; evansimeonidis@gmail.com (E.N.S.); nsofikit@uoi.gr (N.S.); 3 Department of Urology, Faculty of Medicine, School of Health Sciences, University of Ioannina, 45110 Ioannina, Greece; zahariou@otenet.gr; 4 Department of Obstetrics and Gynecology, Royal Cornwall Hospital, Truro TR1 3LJ, UK; athanasios.zikopoulos1@nhs.net; 5 School of Medicine, European University Cyprus, 1516 Nicosia, Cyprus; thaliachrisofou@gmail.com; 6 Department of Urology, Faculty of Medicine, School of Health Sciences, Aristotle University of Thessaloniki, 54124 Thessaloniki, Greece; helabio@yahoo.gr

**Keywords:** endometriosis, transgender men, testosterone therapy, fertility preservation, quality of life

## Abstract

Endometriosis is a debilitating gynecological condition commonly seen in individuals designated female at birth; however, there has been limited research focused on its prevalence and impact among transgender men. This narrative review aims to fill a critical knowledge gap by exploring the epidemiology, clinical manifestations, management strategies, and quality-of-life implications of endometriosis among transgender individuals who identify as male. Specifically, this study seeks to estimate the prevalence rates and describe the symptoms experienced by transgender men undergoing testosterone therapy. Additionally, it addresses the diagnostic challenges posed by hormonal treatments and the lack of culturally competent healthcare services for this population. Recent molecular studies indicate that hormonal imbalances, such as increased estrogen synthesis and progesterone resistance, are significant factors in the persistence of endometriosis symptoms despite testosterone therapy. Moreover, evidence suggests that testosterone therapy may not always suppress endometrial activity completely, contributing to the persistence of symptoms in some individuals. Endometriosis in transgender men requires personalized approaches that consider both testosterone therapy and its interactions with endometriosis, as well as fertility preservation and the psychosocial aspects of treatment. This review emphasizes the necessity of taking an inclusive approach in both research and clinical practice to improve healthcare outcomes for this underserved population. The results demonstrate how continued research, education, and healthcare services tailored specifically to transgender men are necessary to better understand and treat endometriosis, thus improving both their overall health and quality of life.

## 1. Introduction

Endometriosis is a persistent gynecological condition in which tissue similar to the uterine lining appears in other areas of the body, often reacting to hormonal changes and leading to symptoms such as pelvic discomfort, excessive monthly flow, and infertility [1]. At present, it affects an estimated 10% of the global female population, which represents roughly 176 million women worldwide [2].

Endometriosis is widely acknowledged as a disorder affecting cisgender women. Unfortunately, there is limited information and understanding regarding its prevalence and impact among transgender men, presenting unique healthcare challenges. Endometriosis research has predominantly focused on cisgender women, but recent evidence indicates that transgender men also suffer from the condition, with 25% reporting relevant symptoms [3].

Transgender individuals face unique healthcare challenges, particularly regarding endometriosis, due to limited information and understanding of its prevalence and impact. They may experience greater psychological discomfort due to endometriosis symptoms and gender dysphoria symptoms combined. Menstruation symptoms can be highly distressing and increase feelings of gender discordance, compounding an already heavy psychological burden for transgender males. A survey conducted by the National Center for Transgender Equality discovered that 33% of transgender individuals reported negative experiences with healthcare providers, while 23% avoided receiving necessary medical care due to fear of discrimination [4].

Many transgender males face substantial obstacles when accessing culturally incompetent healthcare, complicating diagnosis and treatment. Healthcare practitioners should bridge research gaps and disparities by providing informed and equitable treatments tailored to transgender individuals affected by endometriosis [5]. Due to a lack of research on this condition in transgender people, developing tailored treatment methods and specific recommendations for care remains challenging. Gaining knowledge about its incidence, symptoms, and consequences is integral in providing comprehensive healthcare coverage for this demographic [6].

This narrative review aims to address this gap by exploring the epidemiology, clinical manifestations, management strategies, and quality-of-life implications of endometriosis among transgender men. By analyzing the current literature, the goal is to provide a comprehensive understanding and promote further research to improve treatment outcomes. Specifically, this study seeks to estimate the prevalence rates and describe the symptoms experienced by transgender men undergoing testosterone therapy. Additionally, it addresses the diagnostic challenges posed by hormonal treatments and the lack of culturally competent healthcare services for this population. Through an analysis of the current literature, this review aims to provide a complete picture of this condition within the transgender population as well as promote further research to improve quality treatment outcomes and overall results for those affected by this disease.

Table 1 provides a clear and concise summary of the key aspects and findings related to endometriosis in transgender men compared to cisgender women, as discussed in this narrative review.

## 2. Methods

### 2.1. Literature Search Strategy

PubMed, Scopus, and Google Scholar were searched to retrieve the literature published between January 2003 and May 2024 using keywords such as “endometriosis”, “transgender health”, and “transmasculine individuals”, plus hormone therapies such as testosterone replacement therapy or hormone-deprivation therapy for fertility preservation, fertility preservation management of endometriosis treatment barriers, and quality of life among transgender men.

### 2.2. Inclusion and Exclusion Criteria

#### 2.2.1. Inclusion Criteria

1. Peer-reviewed articles published between 2003 and 2024.
2. Studies focusing on endometriosis and its management.
3. Research discussing endometriosis in transgender individuals.
4. Articles addressing healthcare barriers faced by transgender individuals.
5. Reviews, case studies, clinical trials, and meta-analyses related to endometriosis and transgender health.

#### 2.2.2. Exclusion Criteria

1. Non-English publications.
2. Studies not focusing on the intersection of endometriosis and transgender health.
3. Articles lacking full-text access.
4. Publications before 2003 unless they provide foundational knowledge.

### 2.3. Data Extraction and Synthesis

Data were independently extracted by two reviewers of each selected study, including its design, sample size, population characteristics, key findings, and conclusions. Any discrepancies were discussed and resolved through consensus agreement. Synthesis of the findings from each included study was conducted to provide an extensive snapshot of current knowledge regarding endometriosis among transgender men, including prevalence rates, symptoms, clinical manifestations, the effects of testosterone therapy on the diagnosis, challenges associated with the diagnosis, and management strategies.

### 2.4. Quality Assessment and Limitations

Quality evaluation was carried out for each included study by considering its design, sample size, and relevance to review objectives. The potential limitations of this review include publication bias, limited longitudinal data, and small sample sizes in some studies. Future research should target larger populations that are more diverse with prospective studies that aim to better understand the progression and management of endometriosis among transgender men.

## 3. Epidemiology of Endometriosis in Transgender Individuals

Emerging evidence indicates that transgender men also grapple with endometriosis, thus broadening our understanding of this condition’s scope beyond conventional gender boundaries [5,16]. Research indicates that transgender individuals frequently encounter barriers in accessing healthcare, contributing to the underdiagnosis and underreporting of endometriosis [17]. This uncertainty in medical research and healthcare settings leads to significant gaps in understanding the true prevalence of endometriosis among transgender men [16]. These studies typically utilize retrospective reviews of medical records and surveys of transgender individuals about their symptoms and healthcare experiences, both of which provide valuable insights. While these methods are beneficial, more systematic prospective studies should be conducted to accurately determine prevalence and symptomatology.

Research highlights that the estimated pooled prevalence of endometriosis in transgender men is 25.14%, with a 95% confidence interval from 17.24% to 33.94%. Symptoms such as dysmenorrhea (70.58%), chronic pelvic pain (50.7%), and irregular menstrual cycles (14.9%) are notably prevalent among those not using other medication alongside testosterone [7,8]. Among those who had hysterectomies, 89.5% were taking testosterone, 59.7% had ceased menstruating, and 43.2% experienced painful menstruation. Additionally, the reported stages of endometriosis among those who underwent hysterectomy were primarily stage 1 (40%) and stage 2 (32%), underscoring the varied clinical manifestations of the condition [5].

Managing endometriosis in transgender men presents nuanced challenges that necessitate sensitive diagnostic and therapeutic approaches. Transgender men often face numerous barriers to diagnosis, including social stigmatization, discrimination, and culturally incompetent healthcare providers who fail to acknowledge the needs of gender-diverse people. Reports indicate exposure to mistreatment, such as gender insensitivity, discomfort, denied services, substandard care, verbal abuse, and forced care in healthcare environments [18]. Furthermore, many providers may lack training in recognizing or managing gynecological conditions affecting transgender men, resulting in misdiagnosis or delayed diagnosis [19]. Diagnosis is further complicated by systemic issues such as stigma, discrimination, and a lack of culturally competent healthcare providers, which hinder the accurate identification and treatment of endometriosis in this population [6]. Data indicate that endometriosis is diagnosed in 32% of transgender men who report pelvic pain, compared to 22% of those without pain complaints, illustrating the clinical and diagnostic challenges faced [20].

By addressing these diagnostic challenges and advocating for inclusive research practices, healthcare providers can enhance support for transgender men affected by endometriosis. A deeper understanding of its epidemiology is essential for developing targeted interventions, improving diagnostic protocols, and enhancing healthcare quality for this underserved demographic.

## 4. Clinical Presentation and Diagnosis

### 4.1. Pathophysiology

Endometriosis has an unclear etiology, with various theories proposed as potential pathogenetic mechanisms, though none fully account for all instances of the condition. Sampson’s retrograde menstruation theory proposes that endometrial tissue travels backward through fallopian tubes during menstruation to implant and proliferate in nearby tissues [21]. It accounts for most instances of peritoneal and ovarian endometriosis, as well as cases before menstruation [22] or those associated with Mayer–Rokitansky–Kuster–Hauser syndrome [23]. Additionally, it cannot account for rare cases of male endometriosis either [24].

Another hypothesis includes the coelomic metaplasia theory, which states that serosal tissues connected with the Mullerian ducts may transform into endometrial-like tissue over time [25]. Furthermore, the embryogenetic theory states that endometrial tissue may form from embryonic remnants exposed to estrogen [26]. The stem cell hypothesis asserts that both endometrial and hematopoietic stem cells can differentiate into endometrial cells, potentially leading to endometriotic lesions in various anatomical sites [27]. Endometriosis has often been linked to cancer in its spreading capabilities; endometrial cells travel through both blood and lymph vessels before reaching their destination in organs or structures like pelvic organs [28]. The immune dysregulation theory proposes that flawed apoptotic processes and an inadequate immune response to endometrial cells contribute to endometriosis in unique populations, such as transgender men [29,30].

However, further research must be carried out to fully comprehend endometriosis mechanisms in these unique groups. These insights are crucial for understanding the pathophysiology of endometriosis in transgender men. Research on transgender males who have undergone hysterectomy shows conflicting results regarding endometrial activity. Grimstad et al. found active endometrial tissue in most pathology reports from transgender males who had hysterectomies as part of their gender affirmation process while receiving testosterone [31]. In contrast, Khalifa et al. observed endometrial alterations indicative of an inactive endometrium in a similar group [32]. These conflicting results complicate the understanding of testosterone’s effect on the endometrium. However, the presence of an active endometrium in some patients indicates that not all individuals undergoing testosterone treatment experience the complete suppression of ovarian function and/or endometrial activity.

These divergent outcomes may be attributed to the peripheral conversion of exogenous testosterone into estradiol, a process known as aromatization. Increased androgen levels could potentially convert to estrogen, leading to higher estrogenic activity [33]. This suggests that testosterone therapy alone may not be sufficient to completely suppress endometrial activity in all transgender men. Recent studies elucidate the underlying molecular mechanisms of endometriosis among transgender men undergoing hormonal treatments for their condition. Progesterone receptor ligands such as medroxyprogesterone acetate and dienogest have demonstrated great effectiveness in decreasing endometrial activity by modulating estrogen receptors and decreasing local estradiol production [34]. Epigenetic abnormalities often reduce progesterone receptor expression in endometriotic lesions, contributing to reduced progesterone receptor expression and resistance. Oxidative stress and altered gene expression also exacerbate this resistance; research indicates that targeted therapies, including selective estrogen receptor modulators and aromatase inhibitors, may provide additional benefits by targeting these molecular disruptions [35].

**Figure 1:**
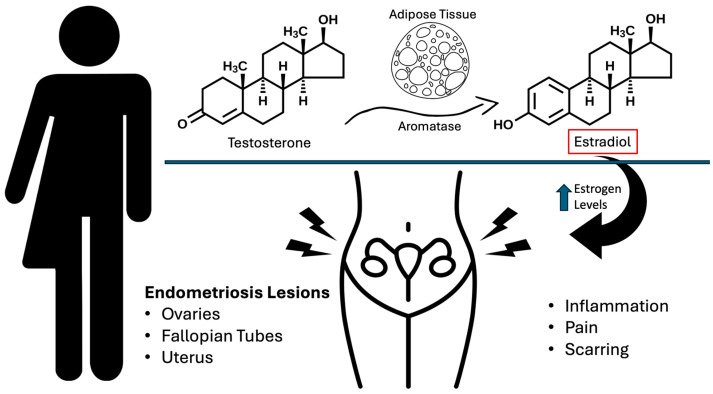
Effects of testosterone treatment and conversion to estrogen in transgender men.

Endometriosis is traditionally considered an estrogen-driven disorder, implicating hormone fluctuations as a key factor in its development. While it remains unclear why transgender individuals exhibit higher rates of endometriosis compared to cisgender individuals, it is hypothesized that elevated androgen levels from long-term testosterone therapy may convert to estrogen, thereby increasing estrogenic activity and potentially contributing to the progression of endometriosis [33]. This could explain the continued symptomatic presentation of endometriosis in transgender individuals even after the cessation of menstruation. This conversion process and its implications for endometriosis are depicted in Figure 1.

At present, there are insufficient data to assess the risk of endometrial disease among transgender males using testosterone, making it impossible to evaluate the risk accurately [36,37]. As a result, routine surveillance measures such as ultrasound or biopsy, as well as prophylactic surgical interventions like hysterectomy, cannot be justified by evidence-based expert opinions [36,37]. Furthermore, its effect as part of gender-affirming hormone therapy remains poorly understood, with its specific effects yet unexplored [36]. Transgender males retaining their ovaries while taking testosterone therapy may experience increased estrogen levels that could contribute to a proliferative endometrium [38]. This situation highlights the complex interplay between hormonal therapy and endometrial health in transgender individuals, emphasizing the need for further research in this area to better understand and manage risks associated with hormone therapy.

### 4.2. Diagnosis

Diagnosing endometriosis among transgender men can be challenging due to the complex interaction between testosterone therapy and endometrial pathology. Studies have demonstrated that testosterone treatment may result in persistent endometrial activity and the appearance of active lesions [39], suggesting that not all individuals experience the complete suppression of endometrial function. Diagnostic approaches should consider hormone levels as well as the peripheral conversion of testosterone to estradiol by using molecular diagnostic tools such as progesterone receptor isoforms that can enhance diagnostic accuracy and determine treatment strategies.

Transgender men who were assigned female at birth but identify as male frequently face challenges when dealing with a healthcare system that does not effectively meet their special needs [40]. Endometriosis, often seen as a mostly female concern, exemplifies this neglect. By acknowledging and examining endometriosis in transgender males, healthcare providers can deconstruct the binary comprehension of this ailment and ensure more comprehensive and accommodating healthcare procedures.

Due to testosterone hormone treatment and the possibility of undergoing surgical procedures like hysterectomy and oophorectomy, the symptoms and manifestations of endometriosis in transgender men may differ from those experienced by cisgender women [41]. Understanding the clinical manifestation and diagnostic difficulties in transgender populations is crucial for providing effective care and support. Healthcare practitioners should be diligent in recognizing endometriosis as a possible reason for pelvic discomfort in transgender males and implement an appropriate diagnostic approach [5]. Endometriosis is often viewed as a condition primarily affecting cisgender women [33]. This limited perspective may lead healthcare practitioners to overlook endometriosis as a possible diagnosis in transgender males. Raising awareness and clinical suspicion among healthcare providers is crucial for recognizing endometriosis in this group.

Diagnosing endometriosis in transgender males can be further complicated by atypical symptoms and a lack of culturally competent healthcare, leading to significant diagnostic delays. Patients may face condescending attitudes or misattribution of their symptoms, resulting in prolonged distress and a diminished quality of life. To overcome diagnostic barriers, healthcare practitioners need to adopt a proactive, open-minded approach and ensure culturally competent and inclusive care practices, which are essential for timely and accurate diagnoses [42].

Testosterone medication, often used during gender transition, may impact/affect the symptoms and manifestation of endometriosis in transgender males [43]. Testosterone-induced hormonal changes might obscure or modify common symptoms, such as alterations in menstrual cycles or pelvic discomfort [44]. Healthcare personnel should be aware of this wide spectrum of clinical manifestations to guarantee precise diagnoses.

There are limited and conflicting data regarding pelvic organ abnormalities in transgender males who have undergone hysterectomy [45]. A previous study analyzed the attributes of uterine abnormalities in 94 transgender males who received testosterone therapy and had a history of hysterectomy as a component of their gender affirmation process [31]. It is worthy of note that the majority of the pathology reports showed the existence of a functioning endometrium in these individuals. On the contrary, other research has examined comparable patient groups and demonstrated that the majority of the samples analyzed had endometrial alterations indicative of an inactive endometrium [32,46].

The intersection of gender identity and gynecological health necessitates a nuanced approach to recognizing and addressing symptoms of endometriosis in transgender individuals. The conflicting results make it difficult to conclusively establish the impact of testosterone on the endometrium. Based on the research, it may be deduced that some patients still have an active endometrium while on testosterone treatment, indicating that their ovarian function and/or endometrial activity is not completely paused. Therefore, transgender males may continue to suffer from the condition even while receiving testosterone therapy.

## 5. Management and Treatment

Providing appropriate education to healthcare professionals and raising clinical suspicion is essential to effectively meeting the medical needs of transgender individuals and treating endometriosis effectively. Education and inclusive care play a vital role in ensuring the appropriate management of endometriosis in transgender individuals. Insufficient expertise among medical personnel in this field may lead to misdiagnosis, delayed treatment, or disregard of symptoms, with a detrimental effect on patients [47]. By reducing this educational disparity, it can be guaranteed that transgender males receive proficient and empathetic treatment from healthcare professionals who comprehend the intricate overlap between their gender identification and endometriosis.

Providing the optimal management of endometriosis among transgender men requires an in-depth knowledge of hormonal influences and molecular pathways, including their potential interactions. Progestins, such as norethindrone acetate and dienogest, are effective in relieving pain by inducing atrophy of the endometrial tissue. They are usually well tolerated and can be administered long-term, making them a preferred first-line treatment [48]. Selective estrogen receptor modulators (SERMs) may help by targeting estrogen-dependent pathways to ease symptoms. Research highlights the significance of maintaining progesterone receptor functionality and mitigating oxidative stress to improve treatment outcomes [34]. GnRH agonists suppress the production of estrogen by downregulating the hypothalamic–pituitary–gonadal axis, inducing a pseudomenopausal state. Although effective in reducing pain, they can have significant side effects, like hot flashes, bone density loss, and mood changes, often necessitating ‘add-back’ therapy with low doses of progestins [49]. Experimental therapies targeting intracellular kinases and inflammatory pathways have also been studied as potential options to treat hormone-resistant endometriotic lesions [50]. Such advanced therapeutic strategies provide promising results for more effective management of endometriosis among transgender men in the future. Combined oral contraceptives (COCs) are another option, suppressing ovulation and stabilizing hormonal fluctuations to reduce dysmenorrhea and pelvic pain. They can be used continuously to avoid menstruation, which is beneficial for transgender men experiencing gender dysphoria related to menstruation. Aromatase inhibitors reduce local estrogen production within endometriotic lesions and are particularly useful for patients not responding to other hormonal therapies, though their long-term use is limited due to potential side effects like bone density loss [51].

The optimal treatment of endometriosis in transgender males requires tailored therapeutic strategies [33]. Hormone treatment, essential for gender transition, might affect the development and symptoms of endometriosis [52]. Healthcare practitioners must have a thorough understanding of the possible connections between testosterone therapy and endometriosis. Medical and surgical options are key components of managing endometriosis in transgender individuals. They should aim to create treatment regimens that achieve a balance between providing care that affirms an individual’s gender identity and reducing symptoms associated with endometriosis. The selection of treatment modalities should be individualized based on the severity of symptoms, the individual’s overall health, and their gender-affirming goals

Surgical interventions play a crucial role. Laparoscopy is the gold standard for diagnosing and treating endometriosis, allowing the direct visualization and excision or ablation of lesions. It is particularly useful for patients with deep infiltrating endometriosis causing significant pain or involving the bowel or urinary tract [53]. A hysterectomy can serve as both a gender-affirming surgery and a definitive treatment for endometriosis, especially when hormonal treatments are ineffective or there is a risk of endometrial carcinoma [33]. Following surgical interventions, postoperative hormonal therapy can help reduce the risk of recurrence and manage residual pain. A combination of surgery and long-term hormonal therapy is often necessary to maintain symptom relief [54]. Transgender men have a higher occurrence of endometriosis compared to cisgender women [7]. Therefore, it is essential for surgeons to conduct a meticulous evaluation of endometriotic foci during surgery in transgender males. However, there is currently a scarcity of data, and more research is required in the future. Support networks and advocacy organizations are essential in empowering transgender males who have endometriosis. These groups may provide essential assistance to transgender males dealing with endometriosis by promoting a feeling of community, increasing awareness, and granting access to resources. Furthermore, by including the perspectives of transgender males in the development of policies and healthcare standards, it can be guaranteed that their distinct experiences and needs will be considered [55].

Healthcare providers must be knowledgeable about the unique healthcare needs and experiences of transgender individuals to deliver culturally competent care [56]. Providing education on endometriosis, its impact on transgender individuals, and available treatment options is essential for empowering patients to make informed decisions about their healthcare [57]. Inclusive care practices, such as using gender-affirming language, respecting individuals’ gender identities, and creating safe and welcoming healthcare environments, are crucial for building trust and promoting positive health outcomes [58]. The importance of multidisciplinary care should be highlighted in the management of endometriosis in transgender individuals. The collaborative engagement of gynecologists, endocrinologists, mental health professionals, and other healthcare providers is essential for addressing the complex needs of transgender individuals with endometriosis [59]. Additionally, involving patients in shared decision-making processes and treatment planning can enhance treatment adherence and patient satisfaction [60].

By integrating medical and surgical interventions, providing education, and fostering inclusive care practices, healthcare providers can optimize the management of endometriosis in transgender individuals. Empowering transgender individuals to actively participate in their care and ensuring that their unique healthcare needs are met are essential steps toward improving the quality of life and health outcomes for this underserved population.

## 6. Impact on Quality of Life

**Figure 2:**
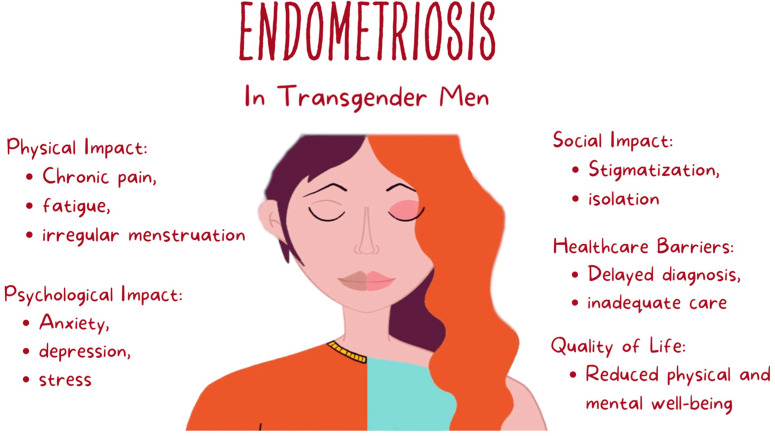
Impact of endometriosis on various aspects of life among transgender men.

Endometriosis can severely restrict transgender individuals’ quality of life, impacting several areas relating to physical, psychological, and social well-being. Understanding these effects and developing tailored interventions are integral parts of providing holistic care to transgender individuals living with endometriosis. Endometriosis’s physical manifestations—chronic pain, fatigue, and irregular menstruation—can significantly compromise transgender individuals’ well-being [15]. These symptoms may impede daily activities, work performance, and overall quality of life, thus highlighting the need for effective management strategies to relieve physical discomfort and increase functional outcomes [61]. Figure 2 illustrates the impact of endometriosis on various aspects of life among transgender men, highlighting the physical, psychological, and social challenges they face.

Endometriosis poses both physical and psychological barriers for transgender individuals, with its attendant challenges of gender identity and healthcare disparities leading to anxiety, depression, and stress [62]. Addressing the psychological impacts of endometriosis with mental health support, counseling, and coping strategies is of utmost importance for transgender individuals seeking emotional well-being and resilience [63].

Endometriosis among transgender individuals must not be ignored due to its social implications. Transgender individuals living with endometriosis often face stigmatizing treatment from healthcare providers and society, compounding their difficulties [64]. Tactically tailored interventions designed to address the unique social needs of transgender individuals—including social support networks, advocacy efforts, and community resources—are key to creating an enabling environment and improving overall well-being [65].

Transgender individuals living with endometriosis require a holistic approach that considers its physical, psychological, and social effects to live fulfilling lives. Individualized interventions tailored specifically to transgender patients’ needs—such as culturally competent care, mental health support, and social inclusion measures—may help reduce the negative effects of endometriosis on transgender people while simultaneously increasing their overall quality of life [66]. By acknowledging its multidimensional effects and providing tailored interventions that address them efficiently, healthcare providers can support transgender people in managing their health challenges while leading fulfilling lives.

## 7. Fertility Preservation

Endometriosis and its treatment can have significant implications for fertility preservation in transgender individuals, necessitating careful consideration and management strategies to address reproductive health concerns within the context of gender-affirming care. The interplay between gender-affirming treatments, the impact of endometriosis on fertility, and the available preservation techniques must be carefully balanced to support reproductive goals.

Testosterone therapy, a common gender-affirming treatment, can adversely affect ovarian function and reduce fertility potential. However, its effects can be partially reversible, allowing some level of reproductive function to be maintained [67]. Endometriosis in transgender individuals may impede fertility preservation options due to its potential impacts on reproductive organs and functions, as endometriosis-related inflammation and scarring can negatively affect the ovaries, fallopian tubes, and uterus, compromising fertility [20,68]. Endometriosis can significantly impact ovarian reserve due to both the disease itself and surgical interventions such as endometrioma excision. Surgical treatment often results in reduced ovarian reserve, underscoring the need for fertility preservation before surgery [69].

Understanding the specific effects of endometriosis on fertility is vital for initiating consultations about preservation options and making informed decisions about them. Considerations and challenges surrounding fertility preservation for transgender individuals living with endometriosis span far beyond physical aspects to encompass psychological and social elements [70]. Navigating fertility decisions while dealing with endometriosis and gender transitioning can be emotionally draining, emphasizing the need for comprehensive support and counseling services to address all associated reproductive and gender identity concerns [71].

Oocyte cryopreservation is a primary method where oocytes are harvested and frozen for future use. This technique requires ovarian stimulation and egg retrieval, which can be distressing for transgender men due to the association with female reproductive anatomy [72]. Embryo cryopreservation involves fertilizing the retrieved oocytes before freezing, providing a higher success rate per thaw cycle but requiring the involvement of sperm, either from a partner or a donor [73]. Ovarian tissue cryopreservation is an emerging technique that involves freezing ovarian tissue for later transplantation. It can be performed concurrently with gender-affirming surgeries and is promising for those who cannot undergo ovarian stimulation [74]. The invasive nature of fertility preservation procedures can exacerbate gender dysphoria. It is crucial to provide supportive care and use gender-affirming language and practices to mitigate distress during the preservation process [75]. Financial barriers and a lack of access to fertility preservation services are significant challenges. Advocacy for insurance coverage and the wider availability of these services is essential to supporting transgender individuals [76].

Integrating fertility preservation strategies that match individual gender identity, reproductive goals, and overall health needs into informed decision-making is essential to maximizing reproductive results and facilitating informed decision-making processes. Healthcare providers can support transgender individuals in making informed decisions regarding their reproductive health by exploring the effects of endometriosis treatment and gender transition on fertility preservation options. By delivering comprehensive information, personalized care, and access to these options, healthcare providers can aid them in making more informed choices for themselves regarding gender transitioning while still managing endometriosis and maintaining fertility preservation options [77,78].

## 8. Future Directions and Research Needs

Endometriosis among transgender individuals represents an intricate intersection of gynecological health and gender identity, necessitating further research to fill any knowledge gaps and enhance care practices for this undertreated population. By identifying areas for future research and emphasizing the necessity of including transgender individuals in endometriosis studies, advances can be made toward better understanding and managing this condition more efficiently.

Transgender individuals’ knowledge of endometriosis is limited, emphasizing the need for research dedicated to this population [79]. Knowledge gaps still remain regarding the prevalence, clinical presentation, impact on quality of life, and treatment outcomes associated with endometriosis in transgender men [79]. More research should be undertaken in order to better understand the experiences and challenges unique to transgender individuals living with endometriosis, including their effects on disease progression and quality of life [79].

Engaging transgender individuals in endometriosis research is crucial for improving understanding and care practices. By including various gender identities in research studies, healthcare providers and researchers can gain greater insights into the needs and experiences of transgender individuals living with endometriosis [80]. An inclusive approach can lead to tailored interventions, guidelines, and treatment strategies designed specifically to address the complexity of managing endometriosis in the transgender population [6].

Future research on endometriosis among transgender individuals should focus on exploring its multidimensional outcomes, including the physical, psychological, and social components of this condition. Longitudinal studies that track the progression of endometriosis among transgender individuals over time and qualitative investigations of their lived experiences can yield valuable insights into its effects on quality of life and well-being [81]. Collaboration among healthcare providers, researchers, and transgender advocacy groups is also key to creating research agendas that meet the needs of transgender people living with endometriosis [20].

By addressing gaps in current knowledge, involving transgender people in research endeavors, and prioritizing comprehensive and inclusive approaches to studying endometriosis among transgender populations, the field can move forward toward more effective management strategies and improved outcomes for this marginalized group. Future research efforts should seek to increase understanding, foster inclusivity, and drive innovation when caring for transgender individuals with endometriosis.

## 9. Conclusions

Endometriosis in transgender men remains under-studied and poorly understood, prompting healthcare providers and scientists to focus more on this condition. This review emphasizes the unique challenges associated with diagnosing and managing endometriosis among this specific population due to complex interactions between hormonal therapies and endometrial pathology. Persistent endometrial activity despite treatment with testosterone highlights the need for customized diagnostic and therapeutic strategies that consider transgender men’s unique hormonal milieu.

Addressing these challenges requires culturally competent healthcare services and inclusive research practices to ensure accurate diagnoses and effective treatment plans. A balance between current research and clinical practice should be established to improve both health and quality of life among transgender men living with endometriosis.

Studies should focus on understanding the molecular mechanisms underlying endometriosis in transgender men and exploring treatments that address hormone resistance, oxidative stress, and multidisciplinary care approaches tailored specifically to trans individuals’ needs. It is vital to foster inclusive care practices while advocating for targeted research as part of comprehensive healthcare approaches tailored specifically to trans people living with endometriosis.

## Figures and Tables

**Table 1:** Key aspects and findings on endometriosis in transgender men compared to cisgender women.

Aspect	Transgender Men	Cisgender Women
**Prevalence Rates**	25.14% (95% CI: 17.24–33.94%) [7,8]	10–18% [9,10,11]
**Common Symptoms**	- Dysmenorrhea (painful menstruation): 70.58% - Chronic pelvic pain: 50.7% - Irregular menstrual cycles: 14.9% [7,8]	Similar symptoms without the influence of testosterone therapy [1]
**Symptoms Persistence**	- Taking testosterone: 89.5% - Cessation of menstruation: 59.7% - Painful menstruation: 43.2% [5]	Symptoms managed through conventional hormonal treatments [12]
**Healthcare Barriers**	Significant barriers, negative healthcare experiences, avoidance of care due to fear of discrimination [13]	Fewer barriers, though misdiagnosis and delayed diagnosis still occur [14]
**Psychological Impact**	Increased anxiety, depression, and stress due to gender dysphoria triggered by endometriosis symptoms [13]	Significant psychological distress but without the component of gender dysphoria [15]

## Data Availability

Not applicable.
